# Social factors related to the quality of life among older adults in southwestern Poland

**DOI:** 10.1371/journal.pone.0349206

**Published:** 2026-05-15

**Authors:** Antonina Kaczorowska, Małgorzata Kołodziej, Anna Sebastjan, Zofia Ignasiak

**Affiliations:** 1 Institute of Health Sciences, University of Opole, Opole, Poland; 2 Faculty of Physical Education and Sport, Wrocław University of Health and Sport Sciences, Wrocław, Poland; 3 Department of Preclinical Sciences, Pharmacology and Medical Diagnostics, Faculty of Medicine, Wroclaw University of Science and Technology, Wrocław, Poland; Policy Innovation Centre, NIGERIA

## Abstract

**Background:**

The quality of life of older adults is closely linked to functioning in the living environment. This study aims to assess the quality of life of older adults in southwestern Poland and the social factors determining it.

**Methods:**

This cross-sectional study included a voluntary sample of 1108 older adults aged 60‒90. The WHOQoL-BREF questionnaire was used to assess quality of life.

**Results:**

The overall quality of life of the respondents (57%) was good. Men had a better quality of life in the psychological (69.0 ± 9.5 vs. 63.0 ± 6.5, p < 0.001) and environmental (69.0 ± 6.0 vs. 63.0 ± 9.5, p < 0.01) domains compared to women. In comparison, those living in a relationship had a better quality of life in the somatic (75.0 ± 9.0 vs. 69.0 ± 9.0, p = 0.034), social (75.0 ± 12.5 vs. 69.0 ± 9.5, p < 0.001), and environmental (69.0 ± 6.0 vs. 63.0 ± 9.5, p < 0.001) domains. Those with higher education had a better quality of life in the somatic (75.0 ± 6.0 vs. 69.0 ± 9.0 and 69.0 ± 9.0, p < 0.001), psychological (69.0 ± 6.5 vs. 63.0 ± 9.5 and 63.0 ± 6.5, p < 0.001), and environmental (69.0 ± 6.0 vs. 63.0 ± 9.5 and 63.0 ± 6.5, p < 0.001) domains compared to those with secondary and primary education. Respondents in good health had a better quality of life in all domains.

**Conclusions:**

Good quality of life in older adults surveyed is determined by male gender, marital status in a relationship, higher education, good health ratings, and fewer chronic diseases. Our results can guide policymakers, highlighting the fact that good health, fewer chronic diseases, and higher education, translate into a better quality of life. Therefore, there should be improved access to education for all people. And good population health should be prioritized by decision-makers.

**Trial registration:**

ISRCTN platform as 18225729; January 2021.

## Introduction

Aging is a multifactorial process consisting of physical, psychological, and health factors [1]. Healthy aging has been defined as ‘the process of developing and maintaining functional ability that enables well-being in old age’ [2]. Healthy ageing is expected to have an impact on quality of life. Therefore, it is important to understand the determinants of quality of life in a healthy ageing perspective [3,4].

Quality of life is a multidimensional concept, encompassing physical, psychological, social and health aspects and activities of daily living [5]. The quality of life of older adults is closely linked to functioning in the living environment, and its maintenance is essential in an era of demographic change, resulting in an increase in life expectancy and the proportion of older people in the population [6]. Since 2006, there has been a steady increase in the share of older people in the Polish population. In 2005, this share was 17.2%, and reached 26.3% in 2023 [7]. Among older adults, as in the general population, women predominate. The share of women in the older population in 2023 was 58.1% [7]. Due to the aging of the population, there has been an increase in the prevalence of chronic illness and disability [8]. According to the demographic forecast, Poland’s population will become increasingly older by 2060. In parallel to a projected decline in the population to 30.9 million people in 2060, a steady increase in the older adult population is expected [7].

The quality of life and health of older adults should be considered in sociocultural terms [9]. Different countries may have different factors affecting the quality of life of older adults [4]. Researchers assessing the quality of life of older adults have found that older age, female gender, subjective low health assessment, loss of independence, lower education, lower economic status, and chronic diseases are among the factors negatively affecting the quality of life in many countries [10–13]. Poland belongs to the post-communist countries of central and eastern Europe. The childhood, adolescence, and adulthood of older adults in Poland, were shaped during the communist era and therefore in a different cultural and economic context than the lives of older adults from Western European and North American countries. People from Central and Eastern Europe often exhibited less healthy lifestyles [14] and treated physical and mental health less than people from Western countries [14,15]. Respondents aged 50 and older from Eastern European countries were more likely to report poor health than respondents from Northern, Western, and Southern Europe [16]. It is therefore necessary to monitor the quality of life of older adults due to the different social factors related to lifestyle in Central and Eastern Europe compared to Western Europe.

Researchers in Poland are increasingly addressing the topic of quality of life in older people, but many aspects of it are still poorly studied. Research does not unequivocally indicate a relationship between quality of life and the gender and education of respondents [17–19]. Other studies on the quality of life of older people in Poland concerned other aspects, such as age and number of chronic diseases [17], physical activity [17–19], handgrip strength [20], and socio-economic status and general health [21].

Research by European and world authors also covers the relationship between smoking and alcohol consumption and the quality of life of older people [4,22–24], but few such studies concern Polish older adults [4]. The results of these studies are inconclusive. Since smoking and alcohol consumption in Poland also affect people aged 60 and over [25,26], it seems reasonable to investigate the relationship between smoking and alcohol consumption and the quality of life of older adults.

It is believed that monitoring the quality of life of older people is essential, and its improvement is becoming one of the key goals in the field of public health. Measuring the quality of life value and identifying factors associated with it is important for the introduction of preventive measures in a region. This study aims to assess the quality of life of older adults living in their homes in southwestern Poland and the social factors determining it.

## Materials and methods

### Design and settings

This cross-sectional study took place between 2010 and 2016. As part of a project, a study was conducted on the quality of life and health status of adults. The study protocol was approved by the Commission for Ethics of Scientific Research of the University of Health and Sport Sciences in Wroclaw (2009, amended in 2015). The measurements took place in the Laboratory of Biokinetics Research having the Quality Management System Certificate -PIV-EN ISO 9001: 2009 (No. PW 48606-10E). The study complied with the ethical requirements for human experimentation according to the Declaration of Helsinki. Recruitment for the study began on October 5, 2009, and ended on September 30, 2016. Participants were informed about the purpose, methods of the study, the procedures used, and the possibility of withdrawing from the study at any stage. All subjects who participated in the study provided written informed consent. The study did not include strictly medical questions, revealing confidential characteristics about the subjects’ health. The research team assured each participant of the confidentiality of the data. The research was retrospectively registered on the ISRCTN platform as 18225729.

### Subjects

Our research included a voluntary sample of older adults aged 60‒90 from the Lower Silesian Voivodeship in southwestern Poland. Participants volunteered for the study advertised in the local media. Invitations were also sent to senior citizens’ centers. A total of 1562 people registered for the study. Of this group, 340 people did not meet the age criterion, 11 people had medical contraindications, and 103 people did not complete the study. In the end, the study group comprised 1108 people, including 314 men and 794 women, aged between 60 and 90 years.

Inclusion criteria included (1) age 60–90 years, (2) ability to move independently, (3) no medical contraindications, (4) good verbal contact, and (5) voluntary written consent to participate in the study. The ability to move independently and the absence of medical contraindications were essential because the subjects also performed a physical fitness test, the results of which have been published in other papers. Exclusion criteria included (1) cancer, acute trauma, and infection, (2) febrile conditions, (3) recent myocardial infarction, (4) other medical contraindications to physical exercise, and (5) lack of consent to participate in the study.

### Methods

The Polish version of the WHOQoL-BREF questionnaire [27,28] was used to assess quality of life. The scale contains 26 questions. The first two, concerning subjective assessment of quality of life and health assessment, are assessed separately. The following questions are grouped into four domains – somatic, psychological, social, and environmental. The somatic domain includes feeling pain, the need for medical treatment for everyday functioning, having energy, and being satisfied with sleep, ability to perform your daily living activities, and capacity to work. The psychological domain concerns enjoying life, a sense of meaning in life, concentration, acceptance of bodily appearance, satisfaction with oneself and experiencing negative feelings. The social domain includes satisfaction with personal relationships, sex life and support from friends. The environmental domain includes a safety, a healthy environment, having money, availability of information, opportunities for leisure activities, and satisfaction with housing conditions, health services, and transport. Each question is scored on a 5-point scale from 1 to 5, where 1 is the lowest score and 5 is the highest. The exceptions are two questions from the somatic domain concerning pain feeling and the need for medical treatment, and one question from the psychological domain concerning the experience of negative feelings, where the coding is reversed. Domain scores are determined by calculating the arithmetic mean of the questions contained in each domain. The higher the domain score, the higher the quality of life. The raw scores were transformed according to the 0–100 key to correspond to the WHOQoL-100 scale scores [27]. The WHOQOL-BREF has been shown to have good to excellent psychometric properties of reliability and performs well in preliminary tests of validity [28]. According to Jaracz et al. [29], who measured the validity and reliability of the WHOQoL scale in the Polish population, a high validity ranging between 0.62–0.76 for the physical domain, 0.55–0.78 for the psychological domain, 0.68–0.85 for the social domain, and 0.58–0.68 for the environmental domain was found. Acceptable internal consistency was shown, with Cronbach’s alpha coefficients being greater than 0.7 for all domains except for the social domain [29].

Patients also completed a short form regarding age, gender, marital status (the response ‘in a relationship’ identified those who were married or living in a civil partnership), number of children born (for women), education, number of chronic diseases, as well as cigarette smoking and alcohol consumption. Smoking and alcohol consumption were recorded as binary variables (yes/no), without further quantification. Information about self-assessed health was collected via self-reported questionnaires, with participants provided assistance.

The Shapiro-Wilk test verified the distribution of quantitative variables (S1 Table). Due to the lack of normality of distribution for all analyzed variables, non-parametric methods were used. For quantitative variables, the median and quartile deviation (Med ± SQ) assessed with the Mann-Whitney U test. The percentage frequency factors were calculated for the categorical data and the χ2 test of independence was performed; interactions between all categorized variables were checked using log-linear analysis. To assess the differences in quality of life parameters depending on social factors, a difference analysis was performed using the Mann-Whitney U test if the social grouping factor had two categories, or the Kruskal-Wallis test if the grouping factor had more categories. Relationships between quantitative variables such as age, the number of chronic diseases, and the quality of life assessment were assessed using Spearman's rank correlation coefficient (ϱ). The statistical significance of the results was assumed at p < 0.05. All analyses were performed using TIBCO Statistica® 13.3.0 (StatSoft Poland).

## Results

### Characteristic of participants

The quality of life of the respondents (57%) was good. Older age was recorded in men (68.4 ± 3.8 years) than in women (65.6 ± 3.8 years), in unmarried people (67.2 ± 4.3 years) than in those living in a relationship (65.8 ± 3.8 years), in those with higher education (67.5 ± 4.2 years) than with secondary education (65.5 ± 3.8 years), and in those rating their health status neutrally rather (67.2 ± 4.6 years) than well (65.6 ± 3.4 years) and not smoking (66.5 ± 3.8 vs. 64.6 ± 3.4 years).

The somatic domain was differentiated by all other factors in addition to gender. Higher values in this domain were obtained by participants living in a relationship (75.0 ± 9.0 vs. 69.0 ± 9.0, p = 0.034), with higher education (75.0 ± 6.0 vs. 69.0 ± 9.0, p < 0.001), and rating their health status as good (81.0 ± 6.0 vs. 66.0 ± 3.0 and 50.0 ± 9.5, p < 0.001). Those who consumed alcohol (75.0 ± 6.0 vs. 69.0 ± 9.0, p = 0.005) and/or smoked cigarettes (81.0 ± 12.5 vs. 75.0 ± 9.0, p = 0.027) scored significantly higher than others in this domain.

Education, health status, and alcohol consumption differentiated the psychological domain in a similar way to the somatic domain. Additionally, men achieved higher values in psychological domain than women (69.0 ± 9.5 vs. 63.0 ± 6.5, p < 0.001).

The social domain was differentiated only by marital status and health status, indicating higher results in this domain among people in a relationship (75.0 ± 12.5 vs. 69.0 ± 9.5, p < 0.001) and good health (75.0 ± 6.0 vs. 69.0 ± 9.5 and 56.0 ± 15.5, p < 0.001).

In the environmental domain, higher scores were recorded in men than in women (69.0 ± 6.0 vs. 63.0 ± 9.5, p = 0.004), in people in a relationship than in single people (69.0 ± 6.0 vs. 63.0 ± 9.5, p < 0.001), and in people with higher education than with secondary and primary education (69.0 ± 6.0 vs. 63.0 ± 9.5 and 63.0 ± 6.5, p < 0.001). Respondents in good health had a better quality of life in all domains (Table 1, S2 Table).

**Table 1:** Characteristics of participants and differences in quality of life assessments (median ± interquartile deviation).

	Overall	Gender		Marital status		Education			Health			Alkohol consumption		Smoking	
	n = 1108	Men n=314 (28%)	Women n = 794 (72%)	in a relationship n=646 (58%)	single n=462 (42%)	higher n=467 (42%)	secondary n=535 (48%)	primary n= 106 (10%)	good n=630 (57%)	average n=434 (39%)	bad n = 44 (4%)	no n=897 (81%)	yes n=211 (19%)	no n=1035 (93%)	yes n = 73 (7%)
age	66.3 ± 3.9	**68.4 ± 3.8**	**65.6 ± 3.8**	**65.8 ± 3.8**	**67.2 ± 4.3**	**67.5 ± 4.2**	**65.5 ± 3.8**	66.7 ± 4.2	**65.6 ± 3.4**	**67.2 ± 4.6**	69.1 ± 5.9	66.4 ± 3.9	66.1 ± 3.9	**66.5 ± 3.8**	**64.6 ± 3.4**
No. of children	2.0 ± 0.5		2.0 ± 0.5	2.0 ± 0.5	2.0 ± 0.5	2.0 ± 0.5	2.0 ± 0.5	2.0 ± 0.5	2.0 ± 0.5	2.0 ± 0.5	2.0 ± 0.5	2.0 ± 0.5	2.0 ± 0.5	2.0 ± 0.5	2.0 ± 0.5
No. of chronic diseases	3.0 ± 1.5	3.0 ± 1.0	3.0 ± 1.5	3.0 ± 1.0	3.0 ± 1.5	3.0 ± 1.5	3.0 ± 1.5	3.0 ± 1.5	**3.0 ± 1.0**	4.0 ± 1.0	**5.0 ± 2.0**	3.0 ± 1.5	3.0 ± 1.5	3.0 ± 1.5	3.0 ± 1.5
generall quality of life	4.0 ± 0.0	4.0 ± 0.0	4.0 ± 0.0	4.0 ± 0.0	4.0 ± 0.5	4.0 ± 0.0	4.0 ± 0.0	4.0 ± 0.5	4.0 ± 0.0	4.0 ± 0.5	3.0 ± 0.5	4.0 ± 0.0	4.0 ± 0.0	4.0 ± 0.0	4.0 ± 0.0
self-assessment of health	4.0 ± 0.5	4.0 ± 0.5	4.0 ± 0.5	4.0 ± 0.5	4.0 ± 0.5	4.0 ± 0.5	4.0 ± 0.5	4.0 ± 0.5	**4.0 ± 0.0**	**3.0 ± 0.5**	**2.0 ± 0.3**	4.0 ± 0.5	4.0 ± 0.5	**4.0 ± 0.5**	**3.0 ± 0.5**
somatic domain	75.0 ± 9.0	75.0 ± 6.0	75.0 ± 9.0	**75.0 ± 9.0**	**69.0 ± 9.0**	**75.0 ± 6.0**	**69.0 ± 9.0**	**69.0 ± 9.0**	**81.0 ± 6.0**	**66.0 ± 3.0**	**50.0 ± 9.5**	**69.0 ± 9.0**	**75.0 ± 6.0**	**75.0 ± 9.0**	**81.0 ± 12.5**
psychological domain	63.0 ± 6.5	**69.0 ± 9.5**	**63.0 ± 6.5**	63.0 ± 6.5	63.0 ± 6.5	**69.0 ± 6.5**	**63.0 ± 9.5**	63.0 ± 6.5	**69.0 ± 9.5**	**56.0 ± 9.5**	**56.0 ± 12.5**	**63.0 ± 6.5**	**69.0 ± 6.5**	63.0 ± 6.5	63.0 ± 6.5
social domain	69.0 ± 9.5	69.0 ± 9.5	75.0 ± 9.5	**75.0 ± 12.5**	**69.0 ± 9.5**	75.0 ± 9.5	69.0 ± 9.5	75.0 ± 12.5	**75.0 ± 6.0**	**69.0 ± 9.5**	**56.0 ± 15.5**	69.0 ± 9.5	75.0 ± 12.5	69.0 ± 9.5	75.0 ± 12.5
environmental domain	69.0 ± 9.5	**69.0 ± 6.0**	**63.0 ± 9.5**	**69.0 ± 6.0**	**63.0 ± 9.5**	**69.0 ± 6.0**	**63.0 ± 9.5**	**63.0 ± 6.5**	**69.0 ± 6.0**	**63.0 ± 6.5**	**59.5 ± 9.5**	69.0 ± 9.5	69.0 ± 6.0	69.0 ± 9.5	69.0 ± 9.5

Note: n- group size, statistically significant differences at p < 0.05 are marked in bold.

Basic statistics of women and men can be seen in S3 –S5 Tables.

### Main results

The relationships between the categorized variables were checked using log-linear analysis. Only two-way interactions were found to be significant for gender, marital status, education, health status, alcohol consumption, and cigarette smoking. No higher-order interactions were demonstrated. The statistics of the most favorable log-linear model are presented in Table 2. The frequencies estimated based on the obtained model did not differ significantly from the observed frequencies (p = 0.914).

**Table 2:** Assessment of partial and marginal relationships for the model including interactions between gender, marital status, education, health status, alcohol consumption and cigarette smoking.

Effect	df	Partial correlation		Marginal correlation	
		χ^2^	p	χ^2^	p
Gender x marital status	1	88.19	<0.001	85.16	<0.001
Gender x education	2	25.28	<0.001	28.80	<0.001
Gender x alcohol consumption	1	35.25	<0.001	42.34	<0.001
Marital status x health status	2	8.13	0.017	9.51	0.009
Education x health status	4	21.59	<0.001	23.32	<0.001
Alcohol consumption x cigarette smoking	1	20.23	<0.001	23.72	<0.001
Model fit	124	103.1	0.914		

Note: χ^2^ - χ^2^ test value, p – statistical significance, x denotes the interaction between two variables,

The prevalence of each category of marital status, education, and alcohol consumption was associated with gender (Table 2). Detailed analysis of these interactions (results not reported in the table due to divisions according to many categorical variables, which reduces their readability) revealed that 81% of men and 49% of women were married/partnered (χ2 = 91.97, p < 0.001), more than half of men (55%) had a higher education, while more than half of women (53%) had a secondary education (χ2 = 30.88, p < 0.001). Those with less than secondary education comprised 9–10% of both gender groups. Alcohol consumption was observed by twice as many men as women (31% vs 14%, χ2 = 42.07, p < 0 .001), while cigarette smoking was identified in 6–7% of participants regardless of gender. Furthermore, a significant interaction between alcohol consumption and smoking was observed, with 77% of participants reporting neither alcohol consumption nor smoking, and 2% using both (χ2 = 13.92, p < 0.001). The majority of participants rated their health status as good irrespective of gender (poor health was indicated by only 4% of participants). Gender did not differentiate the number of chronic diseases present, with only 7% of participants indicating no chronic diseases and one in four participants indicating the presence of at least five different chronic diseases.

The relationship between health assessment and marital status and education was such that good health status was more frequently reported by those in a relationship than those who were single (61% vs 52%, χ2 = 9.27, p = 0.010) and by those with higher education than those with secondary and primary education (63% vs 54% and 43% respectively, χ2 = 19.03, p = 0.008).

Of the women surveyed, 92% were mothers, of whom 31% had 1 child, 53% two, and 16% three or more. The number of children correlated with current marital status (χ2 = 35.61, p < 0.001) and education (χ2 = 75.57, p < 0.001). Having two or more children was more common among women in a relationship (57%), while having one or no children was more common among single women (60–75%). More women with a higher education (45%) compared to the rest (34%) had no children or only one child. The number of children was not associated with self-rated health or substance abuse.

Spearman’s rank correlation coefficient was used to calculate associations between subjective quality of life scores in various domains and participants’ actual socio-demographic conditions. Only the number of chronic diseases and somatic domain scores correlated very weakly with age. The number of chronic diseases was negatively associated with quality of life, health scores, and all domains, with the somatic domain being the strongest and the social domain the least strong. Quality of life and health scores were positively correlated with each other and all domains. The strongest correlations were between health assessment with the somatic domain and the psychological domain with the environmental domain (Table 3).

**Table 3:** Spearman’s rank correlation coefficients (ϱ).

	age	number of chronic diseases	quality of life	self-assessment of health	somatic domain	psychol domainl	social domain	environm domain
age	1.00							
number of chronic diseases	**0.19** (<0.001)	1.00						
quality of life	−0.03 (0.389)	**−0.19** (<0.001)	1.00					
self-assessment of health	−0.03 (0.261)	**−0.35** (<0.001)	**0.44** (<0.001)	1.00				
somatic domain	**−0.12** (<0.001)	**−0.40** (<0.001)	**0.46** (<0.001)	**0.58** (<0.001)	1.00			
psychol domainl	−0.06 (0.066)	**−0.23** (<0.001)	**0.51** (<0.001)	**0.42** (<0.001)	**0.54** (<0.001)	1.00		
social domain	0.01 (0.733)	**−0.18** (<0.001)	**0.42** (<0.001)	**0.27** (<0.001)	**0.35** (<0.001)	**0.45** (<0.001)	1.00	
environm domain	−0.05 (0.129)	**−0.27** (<0.001)	**0.47** (<0.001)	**0.40** (<0.001)	**0.57** (<0.001)	**0.58** (<0.001)	**0.45** (<0.001)	1.00

Note: statistically significant differences at p < 0.05 are marked in bold, p-values for correlation coefficients are in parentheses

## Discussion

Population aging in European countries is a major demographic, social, and economic problem. In this situation, ensuring good quality of life for older adult citizens is a major policy challenge for many European countries. This study aimed to assess the quality of life of older adults in southwestern Poland and the social factors determining it.

Respondents’ quality of life was assessed as well. The quality of life in all domains was differentiated by health ratings. The higher health status was rated, the better the quality of life scores were. This relationship was expected. This relationship was confirmed by the negative correlations of the number of chronic diseases with the scores of all domains and the overall quality of life. In addition to health status, the environmental domain was differentiated by gender, marital status, and education. Higher values were obtained by men, those living in a relationship, and those with higher education. The somatic domain was similarly differentiated in relation to marital status, education, and health; the psychological domain in relation to gender, education, and health; and the social domain in relation to marital status and health. The results of our study indicate that a good quality of life for the surveyed older adults from southwestern Poland is determined by male gender, relationship status, higher education, a good assessment of health status, and a lower number of chronic diseases.

The higher quality of life of older men compared to women is highlighted in many scientific publications [11,17,30,31]. A reason for the higher quality of life of older men may be gender inequality, which persists throughout most of life, from birth to adulthood. Gender inequality affects women and their quality of life in later years. With some variations, this applies to every nation in the global community. Women have poorer access to education, are paid less at work, and are less likely to work in leadership positions. For older adults, the most visible sign of gender inequality is the disproportionate impoverishment of older women compared to older men [32]. Fortunately, gender inequality has narrowed in recent years, but is still significant in the older population [32]. For example, older European women still have less education than men [33]. Women also seem less content than men in most aspects of their lives [34]. By 2050, female longevity will make women 54% of the global population aged 65 and over [35]. In Poland, on the other hand, the share of women over 60 years of age in 2020 was 58% and will be 55% in 2050 [36], thus decreasing slightly but remaining higher than men. This is why ensuring a good quality of life for older women and men is important.

Higher education and being in a relationship are further factors that improve the quality of life of older adults. This fact has been confirmed by other studies [12,17,37,38]. The level of education is not only a broad expertise but also a greater range of information about how the body functions. Higher education enables a proactive approach to one’s own life and health and a choice of behaviors that will result in maintaining quality of life, independence, and health at a good level. Being in a relationship is another factor that positively influences adults’ quality of life [17,39]. Although some studies do not confirm this association [40]. Our study confirms this in relation to older people from southwestern Poland.

It is noteworthy that self-assessed health status influenced quality of life in all domains. The better the health, the better the quality of life of the respondents. And the higher the number of chronic diseases, the worse the quality of life of older adults, which was expected. Health status thus appears to be a strong determinant of the quality of life of older adults in Poland. This relationship is confirmed by other researchers from Poland and other countries [17,41,42]. Many chronic diseases are associated with impaired mobility and limitations in performing daily activities. Also, pain is common in chronic diseases and limits an older adult’s functioning.

The quality of life of a population should be considered in a sociocultural context. Despite the abundance of data on quality of life in countries with different cultures, living conditions, and low and high incomes, information on the relationship between quality of life and socioeconomic characteristics in post-communist countries is still limited. The literature is dominated by studies on Western European and North American countries [14]. Also, the region of the country can influence the health status and quality of life of the population. The older adults surveyed came from the Lower Silesian Voivodship in southwestern Poland, a region that became part of Poland’s administrative area after the Second World War [43]. The region of western Poland has a higher level of industrialization, a more favorable road network, and a higher state of urbanization compared to eastern Poland and is characterized by further dynamic development. One effect of this is the significantly higher level of education of the inhabitants of Wrocław and its surroundings compared to the inhabitants of the eastern regions [44]. This is probably why the older adults surveyed obtained good quality-of-life scores.

Due to the small number of smokers and alcohol drinkers, we cannot draw strong conclusions about the relationship with quality of life. There are only 73 smokers in the entire study group (1108 subjects), which is too small of a group to generalize and transfer these results to the whole society. The group of people who drink alcohol is slightly larger, but these are people who drink occasionally.

To date, the links between social factors and older people's quality of life still appear to be underestimated in the design and implementation of interventions [40]. Reducing inequalities in older people's quality of life should be a key objective of policy and practice. Understanding the association of quality of life with social characteristics is crucial for the implementation of health policies, programs, and intervention strategies aimed at improving the living conditions and quality of life of older adults [14]. Our results can guide policymakers, highlighting the fact that good health and fewer chronic diseases, as well as higher education, translate into a better quality of life. Therefore, there should be improved access to healthcare and education for all people.

### Strength of the study

The study covers a large group of older adults, so the results obtained can be generalized to the area of southwestern Poland. The study was conducted in person (not via the Internet) with all the people surveyed by the same qualified team of researchers, which makes it possible to assess actual quality of life. The study used standardized questionnaires to assess quality of life, and therefore the results can be compared to studies by other authors in Europe and worldwide.

Health professionals and policymakers consider the search for mediating mechanisms to be important from a public health perspective. Understanding the potential determinants of changes in quality of life has important implications for the design and implementation of good practices promoting healthy lifestyles among older people [45]. Our study highlights the importance of modifiable factors, such as health status, number of chronic diseases and level of education, for the quality of life of the older adult population.

### Limitation

The study also has limitations. These include, among others, the cross-sectional nature of the research, which makes it impossible to track the dynamics of changes in involutionary processes. As this study is cross-sectional, causal relationships between social factors and quality of life cannot be inferred. The older adults surveyed came from one region of Poland (the southwestern area), which may not give a complete picture of the situation in the whole country. However, the fact that the study focuses on the quality of life of participants from one region, will allow us to compare the quality of life and social situation of older populations in other regions. The next limitation is that the sample did not include older people with mobility difficulties and some medical limitations, which could also affect the quality of life results. Additionally, since no random sampling was used and only people volunteering for the study were examined. It can be assumed that more physically and socially active people applied to participate. Also, the large number of women surveyed compared to men may distort the results. Therefore, the study sample may not reflect the entire population of older people from southwestern Poland. Future research on the quality of life of older women and men should also include people with reduced mobility and other medical limitations so that the group in the study can more accurately reflect the state of the older adult population in Poland. The study sample should be randomized. This will make the results obtained on quality of life more complete.

## Conclusion

Good quality of life in older adults surveyed is associated with male gender (only psychological and environmental domain), marital status (somatic, social and environmental domain), higher education (somatic, psychological and environmental domain), a good self-assessment of health, and a lower number of chronic diseases. Older age is only weakly associated with the somatic domain.

Further research is needed into the determinants of quality of life in older adults from different regions to be able to effectively implement measures to improve the living conditions and quality of life of the older adult population.

## Supporting information

S1 Table Shapiro-Wilk test results of women and men. (DOCX)

S2 Table Characteristics in relation to social factors. (DOCX)

S3 Table Women's basic statistics. (DOCX)

S4 Table Men's basic statistics. (DOCX)

S5 Table Descriptive statistics of the study groups. (DOCX)
