# Supplementary material for: Social factors related to the quality of life among older adults in southwestern Poland
Source: PLoS One. 2026 May 15;21(5):e0349206. doi: 10.1371/journal.pone.0349206 (PMC13178891; doi:10.1371/journal.pone.0349206)
Supplement: S1 Table — (DOCX) [file pone.0349206.s001.docx]

**S1 Table. Shapiro-Wilk test results of women and men**

|  | Shapiro-Wilk test Men | | | Shapiro-Wilk test Women | | |
| --- | --- | --- | --- | --- | --- | --- |
| Variable | N | W | p | N | W | p |
| Age | 314 | 0.960398 | 0.000000 | 794 | 0.919975 | 0.000000 |
| No. of children | 0 |  |  | 794 | 0.877989 | 0.000000 |
| No. of chronic diseases | 314 | 0.956138 | 0.000000 | 794 | 0.963444 | 0.000000 |
| Quality of life | 314 | 0.696830 | 0.000000 | 794 | 0.712033 | 0.000000 |
| Selfassessment of health | 314 | 0.823618 | 0.000000 | 794 | 0.815698 | 0.000000 |
| Somatic domain | 314 | 0.951926 | 0.000000 | 794 | 0.966138 | 0.000000 |
| Psychological domain | 314 | 0.966813 | 0.000001 | 794 | 0.970916 | 0.000000 |
| Social domain | 314 | 0.948146 | 0.000000 | 794 | 0.935767 | 0.000000 |
| Environmental domain | 314 | 0.971860 | 0.000008 | 794 | 0.971936 | 0.000000 |

N - number of participants, W - Shapiro-Wilk test value, p - probability of W test error, significant difference with normal distribution at p<0.05
