# Supplementary material for: Social factors related to the quality of life among older adults in southwestern Poland
Source: PLoS One. 2026 May 15;21(5):e0349206. doi: 10.1371/journal.pone.0349206 (PMC13178891; doi:10.1371/journal.pone.0349206)
Supplement: S2 Table — (DOCX) [file pone.0349206.s002.docx]

**S2 Table. Characteristics in relation to social factors**

|  | Gender | |  | Marital status | |  | Education | | |  |  |
| --- | --- | --- | --- | --- | --- | --- | --- | --- | --- | --- | --- |
|  | Men | Women | p- value | in a relationship | single | p- value | higher | secondary | primary | p- value |  |
|  | n=314 | n=794 |  | n=646 | n=462 |  | n=467 | n=535 | n= 106 |  |  |
| age | **68.4 ± 3.8** | **65.6 ± 3.8** | **0.000** | **65.8 ± 3.8** | **67.2 ± 4.3** | **0.001** | **67.5 ± 4.2** | **65.5 ± 3.8** | 66.7 ± 4.2 | **0.001** |  |
| No. of children |  | 2.0 ± 0.5 |  | 2.0 ± 0.5 | 2.0 ± 0.5 | 0.999 | 2.0 ± 0.5 | 2.0 ± 0.5 | 2.0 ± 0.5 | 0.199 |  |
| No. of chronic diseases | 3.0 ± 1.0 | 3.0 ± 1.5 | 0.056 | 3.0 ± 1.0 | 3.0 ± 1.5 | 0.189 | 3.0 ± 1.5 | 3.0 ± 1.5 | 3.0 ± 1.5 | 0.168 |  |
| quality of life | 4.0 ± 0.0 | 4.0 ± 0.0 | 0.093 | 4.0 ± 0.0 | 4.0 ± 0.5 | 0.051 | 4.0 ± 0.0 | 4.0 ± 0.0 | 4.0 ± 0.5 | 0.060 |  |
| self-assessment of health | 4.0 ± 0.5 | 4.0 ± 0.5 | 0.557 | 4.0 ± 0.5 | 4.0 ± 0.5 | 0.209 | 4.0 ± 0.5 | 4.0 ± 0.5 | 4.0 ± 0.5 | 0.247 |  |
| somatic domain | 75.0 ± 6.0 | 75.0 ± 9.0 | 0.100 | **75.0 ± 9.0** | **69.0 ± 9.0** | **0.037** | **75.0 ± 6.0** | **69.0 ± 9.0** | **69.0 ± 9.0** | **0.000** |  |
| psychological domain | **69.0 ± 9.5** | **63.0 ± 6.5** | **0.000** | 63.0 ± 6.5 | 63.0 ± 6.5 | 0.102 | **69.0 ± 6.5** | **63.0 ± 9.5** | **63.0 ± 6.5** | **0.000** |  |
| social domain | 69.0 ± 9.5 | 75.0 ± 9.5 | 0.715 | **75.0 ±12.5** | **69.0 ± 9.5** | **0.000** | 75.0 ± 9.5 | 69.0 ± 9.5 | 75.0 ±12.5 | 0.109 |  |
| environmental domain | **69.0 ± 6.0** | **63.0 ± 9.5** | **0.004** | **69.0 ± 6.0** | **63.0 ± 9.5** | **0.000** | **69.0 ± 6.0** | **63.0 ± 9.5** | **63.0 ± 6.5** | **0.000** |  |
|  | Alkohol consumption | |  | Smoking | |  | Health | | |  |  |
|  | no | yes | p- value | no | yes | p- value | good | average | bad | p- value |  |
|  | n=897 | n=211 |  | n=1035 | n=73 |  | n=630 | n=434 | n=44 |  |  |
|  |  |  |  |  |  |  |  |  |  |  |  |
| age | 66.4 ± 3.9 | 66.1 ± 3.9 | 0.122 | **66.5 ± 3.8** | **64.6 ± 3.4** | 0.002 | **65.6 ± 3.4** | **67.2 ± 4.6** | **69.1 ± 5.9** | **0.000** |  |
| No. of children | 2.0 ± 0.5 | 2.0 ± 0.5 | 0.101 | 2.0 ± 0.5 | 2.0 ± 0.5 | 0.107 | 2.0 ± 0.5 | 2.0 ± 0.5 | 2.0 ± 0.5 | 0.311 |  |
| No. of chronic diseases | 3.0 ± 1.5 | 3.0 ± 1.5 | 0.701 | 3.0 ± 1.5 | 3.0 ± 1.5 | 0.094 | **3.0 ± 1.0** | 4.0 ± 1.0 | **5.0 ± 2.0** | **0.000** |  |
| quality of life | 4.0 ± 0.0 | 4.0 ± 0.0 | 0.101 | 4.0 ± 0.0 | 4.0 ± 0.0 | 0.812 | 4.0 ± 0.0 | 4.0 ± 0.5 | 3.0 ± 0.5 | 0.102 |  |
| self-assessment of health | 4.0 ± 0.5 | 4.0 ± 0.5 | 0.424 | **4.0 ± 0.5** | **3.0 ± 0.5** | **0.016** | **4.0 ± 0.0** | **3.0 ± 0.5** | **2.0 ± 0.3** | **0.000** |  |
| somatic domain | **69.0 ± 9.0** | **75.0 ± 6.0** | **0.016** | **75.0 ± 9.0** | **81.0 ±12.5** | **0.027** | **81.0 ± 6.0** | **66.0 ± 3.0** | **50.0 ± 9.5** | **0.000** |  |
| psychological domain | **63.0 ± 6.5** | **69.0 ± 6.5** | **0.002** | 63.0 ± 6.5 | 63.0 ± 6.5 | 0.773 | **69.0 ± 9.5** | **56.0 ± 9.5** | **56.0 ±12.5** | **0.000** |  |
| social domain | 69.0 ± 9.5 | 75.0 ±12.5 | 0.219 | 69.0 ± 9.5 | 75.0 ±12.5 | 0.456 | **75.0 ± 6.0** | **69.0 ± 9.5** | **56.0 ±15.5** | **0.000** |  |
| environmental domain | 69.0 ± 9.5 | 69.0 ± 6.0 | 0.101 | 69.0 ± 9.5 | 69.0 ± 9.5 | 0.892 | **69.0 ± 6.0** | **63.0 ± 6.5** | **59.5 ± 9.5** | **0.000** |  |

Statistically significant differences at p<0.05 are marked in bold
