# Supplementary material for: Social factors related to the quality of life among older adults in southwestern Poland
Source: PLoS One. 2026 May 15;21(5):e0349206. doi: 10.1371/journal.pone.0349206 (PMC13178891; doi:10.1371/journal.pone.0349206)
Supplement: S5 Table — (DOCX) [file pone.0349206.s005.docx]

**S5 Table.** **Descriptive statistics of the study groups**
